# Unmanipulated bone marrow infusion without conditioning can rescue *RECQL4*^−/−^ severe combined immunodeficiency

**DOI:** 10.70962/jhi.20250169

**Published:** 2026-03-06

**Authors:** Alexandra E. Grier, Marita Bosticardo, Francesca Pala, Ottavia M. Delmonte, Kayla Amini, Eduardo Anaya, Neil Romberg, Susan E. McClory, Nancy J. Bunin, Manar Abdalgani, Luigi D. Notarangelo, Jennifer Heimall

**Affiliations:** 1 Division of Allergy and Immunology, Children’s Hospital of Philadelphia, Philadelphia, PA, USA; 2 Laboratory of Clinical Immunology and Microbiology, National Institute of Allergy and Infectious Diseases, National Institutes of Health, Bethesda, MD, USA; 3 Division of Oncology, Children’s Hospital of Philadelphia, Philadelphia, PA, USA; 4 Department of Pediatrics, Columbia University Vagelos College of Physicians and Surgeons, Columbia University, New York, NY, USA

## Abstract

Mutations in DNA helicase RECQL4 can cause immunodeficiency, including SCID. The T cell defects are intrinsic to the hematopoietic system and amenable to hematopoietic stem cell transplant. Radiosensitivity due to DNA repair defects limits conditioning regimen options.

## Introduction

RECQL4 is a DNA helicase involved in DNA replication, recombination, and repair (1). Mutations in *RECQL4* cause three distinct but overlapping rare syndromes: Baller-Gerold syndrome (BGS), Rothmund-Thomson syndrome (RTS), and RAPADILINO syndrome (RS) (1). All share features of slow growth, radial ray defects, and cancer predisposition. Immune deficiency has not been consistently described as a prominent clinical feature. However, there are several case reports in the literature describing various immune defects in RECQL4 diseases, primarily combined immunodeficiency (2).

Given the wide variety of phenotypes associated with RECQL4 deficiency and the rarity of reported immune defects in these diseases, the optimal treatment approach for severe T cell deficiency in patients with RECQL4-associated disease is unclear. Importantly, it is unknown whether the T cell defects are thymic or hematopoietic in origin. In humans, RECQL4 is highly expressed both in the bone marrow and thymus. Animal models provide conflicting evidence, with some *Recql4* knockout mice demonstrating thymic hypoplasia, while mice with *Recql4* somatic mutations developing bone marrow failure (3). Determining the nature of the T cell defect is paramount, as it determines whether an affected RECQL4-deficient patient would benefit from thymic tissue implant or hematopoietic stem cell transplantation (HSCT). And finally, if HSCT is the recommended treatment, the conditioning strategy will require careful design due to defective DNA damage repair in RECQL4 deficiency.

## Results and discussion

We report a male patient with BGS due to homozygous pathogenic variants in *RECQL4* who was found to have severe T cell deficiency on T cell receptor excision circle (TREC)–based newborn screening, with TRECs >0 but <25 copies/μl. The patient was also noted to have growth restriction and multiple congenital anomalies (including craniosynostosis, limb anomalies, and small thymus) on prenatal ultrasound. After birth, the patient was found to have severe diarrhea requiring parenteral nutrition, ventriculomegaly, respiratory insufficiency, bilateral sensorineural hearing loss, and decreased bone mineralization leading to nontraumatic fractures.

Due to the multiple congenital anomalies noted on fetal scans, prenatal genetic testing was performed. Both parents are of Ashkenazi Jewish ancestry. There is no reported consanguinity. Prenatal whole-genome sequencing demonstrated maternally and paternally inherited homozygous pathogenic splice variants in *RECQL4* (c.2464-1G>C). No pathogenic or likely pathogenic variants were identified in any other immune deficiency-associated genes. This *RECQL4* variant was previously reported as pathogenic in two brothers diagnosed with RTS (4) and is extremely rare in population databases (total allele frequency of 7.96 × 10^−5^ in gnomAD; 0.003128 in the Ashkenazi Jewish ancestry group) with no homozygotes reported. SpliceAI predicts the variant to affect splicing, with an acceptor loss score of 0.98. The variant is predicted to be deleterious, with a Combined Annotation Dependent Depletion score of 22.5 and a 95% mutation significance cutoff score of 5.64.

**Figure 1.**
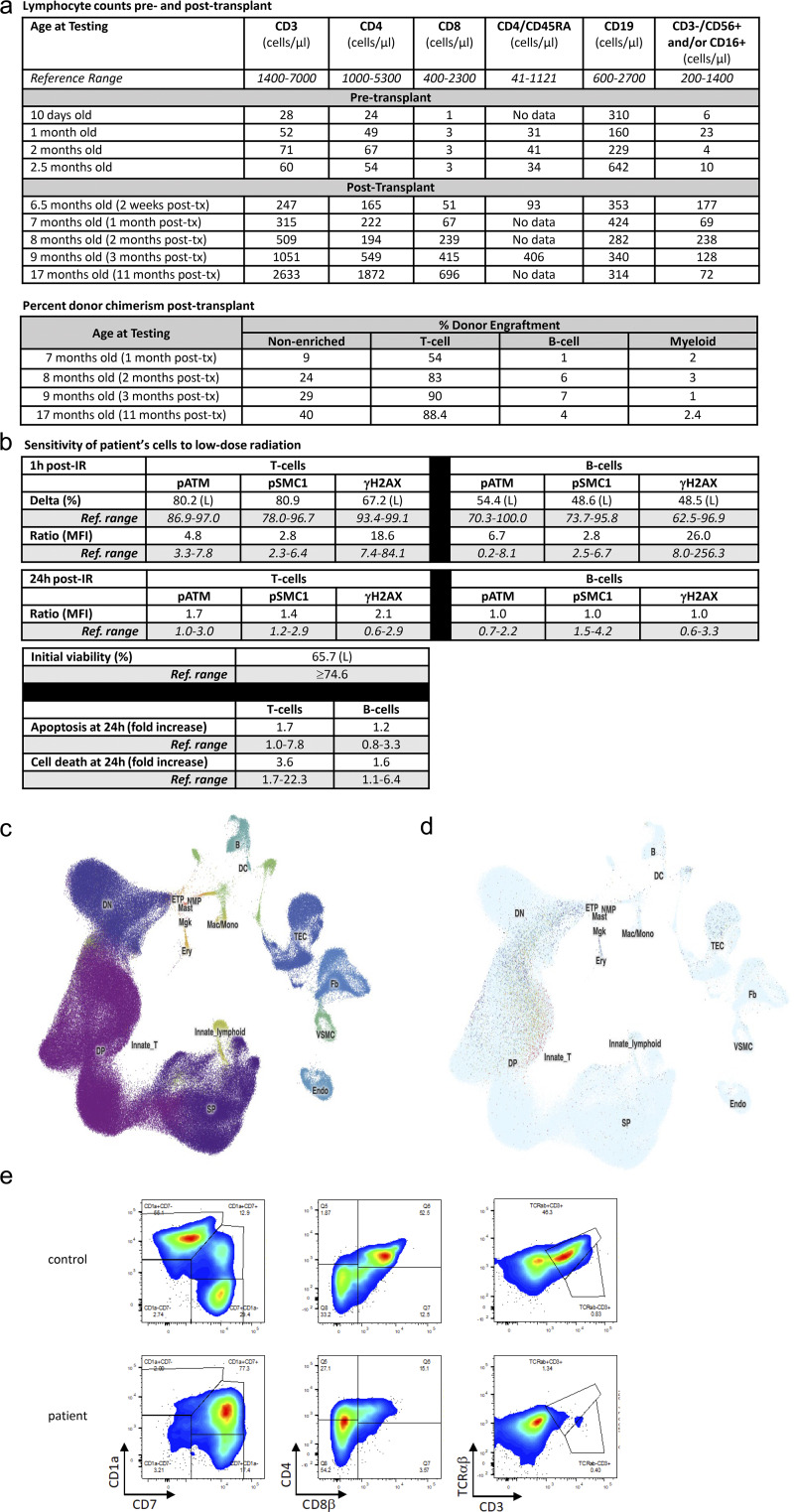
**RECQL4 deficiency is associated with DNA radiosensitivity and a hematopoietic-intrinsic T cell defect than can be rescued with HSCT. (a)** Lymphocyte cell counts pre- and post-transplant and percent donor chimerism after transplant. **(b)** Defective induction and completion of DNA double-stranded break repair in the patient’s T and B cells as measured after exposure to low-dose irradiation in a commercial assay. Too few NK cells were present in the patient sample for analysis. 1 and 24 h after irradiation, the percentage of cells in which each marker (pATM, pSMC, and γ H2AX) was phosphorylated was measured by flow cytometry. The percent of cells phosphorylated is expressed as the difference (delta) between irradiated and unirradiated cells. The degree of phosphorylation is expressed as the ratio of the mean fluorescence intensity of irradiated/unirradiated cells. The viability of total lymphocytes was low at the start of the assay, but there was no significant increase in cell death or apoptosis at 24 h after irradiation, though there may be increased cell death over time, resulting in lymphopenia. **(c)** Uniform Manifold Approximation and Projection visualization showing the cellular composition of the human thymus obtained from the collection of single-cell datasets available on the UCSC Cell Browser (https://fetal-thymus.cells.ucsc.edu). **(d)** Expression of *RECQL4* in the human thymus atlas. RECQL4 is expressed in the human thymus in CD4^−^ CD8^−^ double negative (DN), CD4^+^ CD8^+^ double positive (DP) thymocytes, and in thymic epithelial cells (TECs). **(e)** Defective T cell differentiation from CD34^+^ stem cells in the patient after 7 wk in an ATO system. The ATOs are made by aggregating a human DLL4-expressing stromal cell line (MS5-hDLL4) with CD34^+^ cells isolated from peripheral blood of either the patient or a healthy control. Plots shown are gated on CD45^+^CD56^−^ cells. Data shown are representative of two independent experiments. ETP, early T cell progenitor; MFI, mean fluorescence intensity. tx, transplant; IR = irradiation; DC, dendritic cell; SP, single positive thymocyte; NMP, neutrophil-myeloid progenitor; VSCM, vascular smooth muscle cell.

Initial immunophenotyping in our patient confirmed a diagnosis of likely T^−^B^low^NK^−^ severe combined immunodeficiency (SCID) with T cell counts of 28–71 cells/μl, B cell counts of 160–642 cells/μl, and natural killer (NK) cell counts of 4–23 cells/μl (Fig. 1 a). Approximately 60% of CD4 T cells were naive based on expression of CD45RA (Fig. 1 a). Maternal engraftment testing was negative. A commercial radiosensitivity assay performed due to the known role of RECQL4 in DNA double-strand break repair demonstrated moderate deficiency in both induction and completion of double-strand break repair in T and B cells (Fig. 1 b). As T cell deficiency can be the result of a thymic or hematopoietic defect, publicly available single-cell RNA-sequencing data were queried to define expression patterns of RECQL4 in the human thymus. RECQL4 transcripts were highest in early T cell progenitor cells, double negative and double positive thymocytes (i.e., hematopoietic cells), but were also expressed in a proportion of cortical thymic epithelial cells (Fig. 1, c and d, and additional data not shown, available upon request).

Upon consenting the patient to Protocol 6907 of the Primary Immune Deficiency Treatment Consortium, approved by the Institutional Review Board of Children’s Hospital of Philadelphia, we utilized artificial thymic organoids (ATOs) to experimentally isolate the effects of RECQL4 deficiency in hematopoietic cells. ATOs were used to culture patient CD34^+^ cells into early T cells in the presence of a RECQL4-sufficient, DLL4-expressing stromal cell line (MS5-hDLL4) (5). Compared with healthy control CD34^+^ cells, which developed normally into CD4^+^CD8^+^TCRαβ^+^ thymocytes, patient counterparts arrested at the pre-T1 double negative stage of thymocyte development, with low numbers of CD4^+^ CD8^+^ double positive cells and near complete absence of CD3^+^ TCRαβ^+^ cells (Fig. 1 e). These results confirmed the presence of a hematopoietic-intrinsic defect. HLA typing of the extended family identified that two of his siblings were 12/12 matched donors. Neither sibling carried the *RECQL4* variant.

Due to the radiosensitivity associated with RECQL4 deficiency, the confirmation of a hematopoietic-intrinsic defect, the availability of a matched sibling donor, and the patient’s multiple comorbidities, multidisciplinary discussions took place between immunology, transplant, and medical ethics teams together with the family to consider potential approaches to longitudinal management. While the patient was deemed at too high risk to undergo chemotherapy-based conditioning for HSCT, given the need for long-term subacute support, the decreased survival with delayed HSCT in SCID (6), and the apparent susceptibility of RECQL4-deficient patients to herpesvirus infections (2), it was also deemed ill-advised to take a watch-and-wait approach. Furthermore, given his lack of T cells and NK cells, it was presumed there would be an inability to reject a well-matched graft. Therefore, the patient was treated with unmanipulated bone marrow infusion without conditioning from his matched sibling donor at 6 mo of age. Initial evaluation at 3 mo after transplant demonstrated emerging T cell development (Fig. 1 a) with normal TRECs (14,616 copies/10^6^ T cells; reference range ≥6,794), a polyclonal TCR repertoire via CD3 spectratyping, and 76% of CD4 T cells expressing naive markers. Most recent evaluation at 11 mo after transplant continues to demonstrate normal T cell counts (Fig. 1 a) and nearly full T and NK cell donor engraftment (Fig. 1 a), but, as expected, low B and myeloid-lineage chimerism. T cell reconstitution in patients with SCID who receive unmanipulated matched sibling bone marrow infusions can either be due to post-infusion homeostatic expansion of mature T cells contained in the graft or to naive T cell development from hematopoietic progenitors. In this case, the high proportion of T cells expressing naive T cell markers, the normal TREC numbers, and the polyclonal T cell repertoire all indicate that his T cell reconstitution is due to endogenous production of naive T cells from the graft. He remains on immunoglobulin replacement therapy, but his most recent immunoglobulin measurements demonstrate production of IgM and IgA. It is important to note that HSCT was only able to ameliorate the effects of RECQL4 deficiency in hematopoietic cells; the patient’s skeletal defects, slow growth, developmental delay, and cancer predisposition remain.

To our knowledge, this is the first report of SCID in BGS due to RECQL4 deficiency. Although immune defects are not a prominent feature of any RECQL4-associated diseases, there are four case reports of patients with RECQL4 deficiency and immune defects. Like our patient, all four of the patients previously described had hypogammaglobulinemia, NK cell deficiency, and most had combined immunodeficiency. The first patient was a 2-mo-old female with BGS and isolated humoral defects who died of sepsis (2). The second patient was a 6-mo-old male with RTS who was found to have a combined immunodeficiency (T^−^B^+^NK^−^ phenotype) after developing *Pneumocystis jirovecii* pneumonia. He was definitively treated with a mismatched unrelated cord blood transplant with reduced intensity conditioning with cyclophosphamide (80 mg/kg), fludarabine (120 mg/m^2^), and antithymocyte globulin. His course was complicated by grade IV acute graft-versus-host disease, but he ultimately achieved full donor chimerism by 1 year after transplant (2). The third patient was a 2-year-old female with RTS and low CD8 T cells and NK cells, normal CD4 T cells and B cells, and normal T cell proliferation who developed sinopulmonary infections as well as varicella zoster virus infection with cutaneous granuloma formation (2). The fourth reported patient was a 2-year-old female with RS with low T, B, and NK cells as well as low T cell proliferation who developed nontuberculous mycobacterial granulomas (2). The third and fourth patients were maintained on immunoglobulin replacement therapy and antimicrobial prophylaxis (2).

The full functional biology of RECQL4 and the diseases caused by its deficiency are not well understood, and genotype–phenotype correlations are lacking (7). Although there are overlapping features of BGS, RTS, and RS, each disease also has distinct features in terms of skeletal abnormalities, cutaneous manifestations, and cancer predisposition. Within each disease, there is a wide range of developmental outcomes and other phenotypic features, such as immunodeficiency. RECQL4 has been shown to interact with many other proteins and localize to the nucleus, cytoplasm, and mitochondria (1). The diverse functions, binding partners, and sites of localization of RECQL4 likely account for the varying phenotypes of the associated disorders and the lack of clear genotype–phenotype correlations. Here, we demonstrate *in vitro* with ATOs and *in vivo* through HSCT that the T cell deficiency in RECQL4 diseases is due primarily to a hematopoietic defect. Specifically, the RECQL4 defect leads to an arrest in T cell development at the pre-T II stage, similar to that seen in PTCRA defects. This arrest occurs later in development than is seen in IL2RG or AK2 defects, but earlier than that seen in RAG1/RAG2 defects (data not shown; available upon request). Experiments in animal models and *in vitro* with human cells have demonstrated roles for RECQL4 in many processes such as DNA replication, double-stranded break repair, base excision repair, nucleotide excision repair, mitochondrial genome maintenance, telomere stability, and cross-link repair (1). In other forms of immunodeficiency associated with radiosensitivity, use of alkylator-based conditioning regimens to prepare for HSCT have been associated with increased risks of late effects. Novel approaches to the preparative regimen, particularly for patients with radiosensitive forms of SCID, are in development. These utilize monoclonal antibodies to the stem-cell marker c-kit (CD117) or CD45 antibody-drug conjugates to open broader marrow niches and improve multi-lineage engraftment.
